# FMCW Radar Sensors with Improved Range Precision by Reusing the Neural Network

**DOI:** 10.3390/s24010136

**Published:** 2023-12-26

**Authors:** Homin Cho, Yunho Jung, Seongjoo Lee

**Affiliations:** 1 Department of Semiconductor Systems Engineering, Sejong University, Gunja-dong, Gwangjin-gu, Seoul 05006, Republic of Korea; homin@itsoc.sejong.ac.kr; 2 Department of Convergence Engineering of Intelligent Drone, Sejong University, Gunja-dong, Gwangjin-gu, Seoul 05006, Republic of Korea; 3 Department of Smart Drone Convergence, Korea Aerospace University, Goyang 10540, Gyeonggi-do, Republic of Korea; yjung@kau.ac.kr; 4 School of Electronics and Information Engineering, Korea Aerospace University, Goyang 10540, Gyeonggi-do, Republic of Korea

**Keywords:** FMCW, FMCW radar, range precision, supervised learning, methodology

## Abstract

This paper addresses the challenge of enhancing range precision in radar sensors through supervised learning. However, when the range precision surpasses the range resolution, it leads to a rapid increase in the number of labels, resulting in elevated learning costs. The removal of background noise in indoor environments is also crucial. In response, this study proposes a methodology aiming to increase range precision while mitigating the issue of a growing number of labels in supervised learning. Neural networks learned for a specific section are reused to minimize learning costs and maximize computational efficiency. Formulas and experiments confirmed that identical fractional multiple patterns in the frequency domain can be applied to analyze patterns in other FFT bin positions (representing different target positions). In conclusion, the results suggest that neural networks trained with the same data can be repurposed, enabling efficient hardware implementation.

## 1. Introduction

Radar is a sensor that uses reflected waves from objects to determine the target range. Radar sensors are widely used to obtain various information such as the distance, position, speed, and material type of objects. As the application fields of radar sensors become more diverse, there is a noticeable trend of demanding high performance even in inexpensive radar sensors. Among the performance requirements for low-cost radar sensors, distance accuracy is the most crucial, and various research efforts are needed to enhance this capability. The more accurate the range precision, the higher the possible performance in cars, drones, security facilities, IoT equipment, etc. The accuracy of a target range with FMCW radar is determined by the range resolution, which in turn depends on the bandwidth of the radar waveform. A wider bandwidth leads to higher range resolution, and bandwidth depends on the waveform generated by the oscillator. However, an oscillator with a wide bandwidth incurs high costs. Signal processing algorithms enable the enhancement of radar range resolution while maintaining cost efficiency. In this paper, the terms *range resolution* and *range precision* are used as follows: *range resolution* refers to the minimum unit that can distinguish between multiple targets, indicating different objects. *Range precision* is used as the unit that accurately represents the position of a single target. The most commonly used algorithm is zero padding [1]. Alternative algorithms include the use of n-point mirrors or phase padding on time-domain (TD) samples [2]. Padding can improve the range accuracy of the Fourier transform (FT) by taking more samples within the coherence processing interval (CPI).

Within indoor environments, the noise floor must be considered. This term refers to reflected waves, multipath interference, and signals from objects such as indoor walls and pillars, which can mask the desired signal [3,4,5]. Discontinuities in the time-domain signal lead to phase errors, resulting in clutter noise in the frequency domain and a reduction in the range precision [3,6]. In [7], index-based distance estimation (IBDE) trains the beat frequency pattern associated with each label, effectively reducing range precision errors. It ensures the prevention of discontinuities, even in the absence of background interference. Additionally, there is no need to increase the FFT points since the number of samples does not increase. The IBDE method employs supervised learning which necessitates acquiring and organizing a sufficient amount of data for each label. Consequently, the issue of labeling has been raised, particularly regarding cost, leading to the investigation of various methods [8,9,10]. FMCW radar also encounters limitations owing to the labeling issue. This is because the supervised learning model requires labeling for each beat frequency index. This paper introduces a method to address the labeling concern while efficiently reusing neural networks to enhance range precision in FMCW radar.

Section 2 briefly explains the structure and signals of FMCW. Section 3 introduces the proposed algorithm and explains the utilization of the neural network architecture. Section 4 details the experimental environment and results. Finally, Section 5 presents the conclusions.

## 2. Fundamental FMCW Radar Waveform

**Figure 1:**
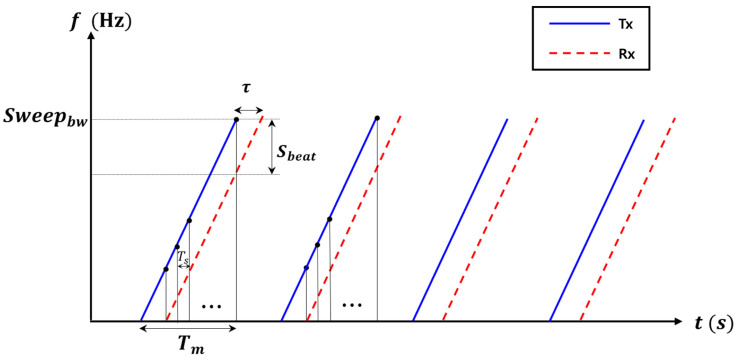
FMCW radar waveform.

FMCW radar uses signals that increase in frequency over time. Figure 1 shows the FMCW sawtooth signal. Tx refers to the transmitted signal and Rx refers to the received signal. τ refers to the round-trip delay time of the target. $f_{beat}$ is the beat frequency, $T_{s}$ is sampling period, and N is the number of FFT points. Additionally, $S_{beat}$ is the beat frequency, $T_{m}$ is the chirp period, $T_{s}$ is the sampling period, and N represents the number of FFT points.

**Figure 2:**
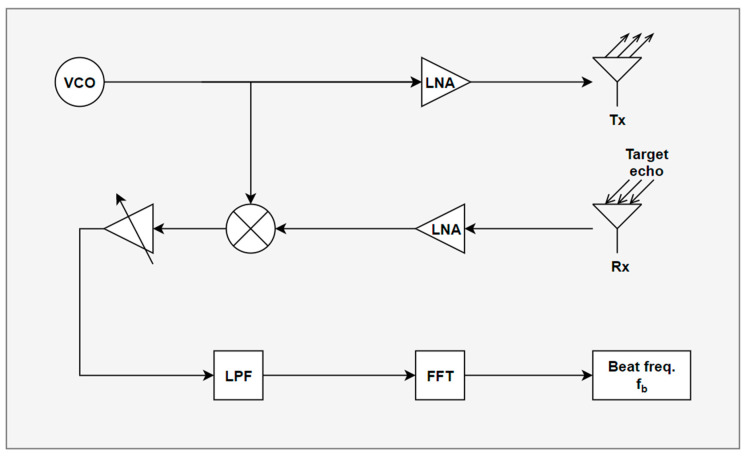
Signal processing flow in FMCW radar.

Figure 2 delineates the signal processing procedure employed to derive the target distance from the radar. The voltage-controlled oscillator (VCO) generates a FMCW waveform, which is subsequently transmitted by Tx after passing through a low noise amplifier (LNA). The signal received by Rx is then mixed with the VCO-generated signal and amplified. Subsequently, the beat frequency is extracted by eliminating the carrier component through a low-pass filter (LPF). The amplitude of the received signal in the frequency domain determines the target range. Equation (1) represents the beat signal post LPF processing, as depicted in Figure 2.

$$s_{beat}\left(t\right)=A^{2}\sigma exp(j2\pi f_{c}\tau +j2\pi {Sweep}_{bw}\tau \Pi (t/T_{m})$$

(1) $$\Pi \left(t/T_{m}\right)=\left(\begin{array}{c} \;1\;\;\;\;\;-\frac{T_{m}}{2}<t<\frac{T_{m}}{2}\\ 0\;\;\;\;\;\;\;\;\;\;\;\;\;\;\;otherwise \end{array}\right)$$

(2) $$S_{beat}\left(f\right)=A^{2}\sigma \exp\left(j2\pi f_{c}\tau \right)sinc\left(T_{m}\left(f-\frac{{Sweep}_{bw}}{T_{m}}\frac{2R}{c}\right)\right)$$

In the radar signal processing, $s_{beat}\left(t\right)$ represents the beat frequency formed by mixing the transmitted and received signals [11]. $S_{beat}(f)$ is the Fourier transform of $s_{beat}(t)$. *A* is the amplitude of the transmitted signal, and σ means the amplitude attenuation attributed to radar cross-section and path loss. The variable *R* denotes the target range.

(3) $$S_{f}\left(n\right)={\left.S_{beat}\left(f\right)\right|}_{f=nFs}$$

(4) $$n_{max}=\begin{array}{c} argmax\\ n \end{array}\left|S_{f}\left(n\right)\right|$$

Equation (3) represents the beat frequency as the fast Fourier transform (FFT) of a discrete frequency signal. Here, *Fs* is defined as 1/*Ts*, where n denotes the FFT bin number. In Equation (4), the parameter $n_{max}$ signifies the argument n at which the magnitude of $S_{f}(n)$ is maximal.

## 3. Proposed Algorithm

In radar signal processing, when the target’s range *R* is an integer multiple of the range resolution, it can be expressed using Equation (5). Applying Equation (5) from Equation (2) results in the expression given by Equation (7). In this paper, we consider the target range by separating it into integer multiples and fractional multiples of the range resolution. The target range ($d_{target}$) can be expressed by Equation (6), where *n* is the integer multiple range, consistent with the *n* used in Equation (5), and γ represents the radar’s range resolution. The parameter *p* defines the fractional multiple range. Substituting this into Equation (2) results in the simplified form presented as Equation (8). It signifies the sinc shift by *K*. When *p* equals zero, *S_f_*(*n*) exhibits zero-crossing sidelobes. Otherwise, for non-zero values of *p*, *S_f_*(*n*) follows a sinc shape, where the sidelobes are dependent on the term *K*. Hence, the sidelobes of *S_f_*(*n*) shifted by p are dependent on the term *K*. That is, the sinc pattern of *S_f_*(*n*) is determined by the term *K* and is independent of *n*.

(5) $$R=n\frac{c}{2{Sweep}_{bw}}$$

(6) $$d_{target}=R+p=n\gamma +p\;\;\;\;\;\begin{array}{c} n=0,\;1,\;2,\;\ldots ,\;N\\ p\;|\;0\leq p<\gamma \end{array}$$

(7) $$S_{beat}(f)=A^{2}\sigma T_{m}\exp\left(j2\pi f_{c}\tau \right)sinc\left(T_{m}\left(f-\frac{n}{T_{m}}\right)\right)$$

(8) $$S_{beat}\left(f\right)=A^{2}\sigma \exp\left(j2\pi f_{c}\tau \right)sinc\left(T_{m}\left(f-(\frac{n}{T_{m}}+K\;)\right)\right),\;\;\;K=\frac{2Sweep_{bw}}{cT_{m}}p$$

**Figure 3:**
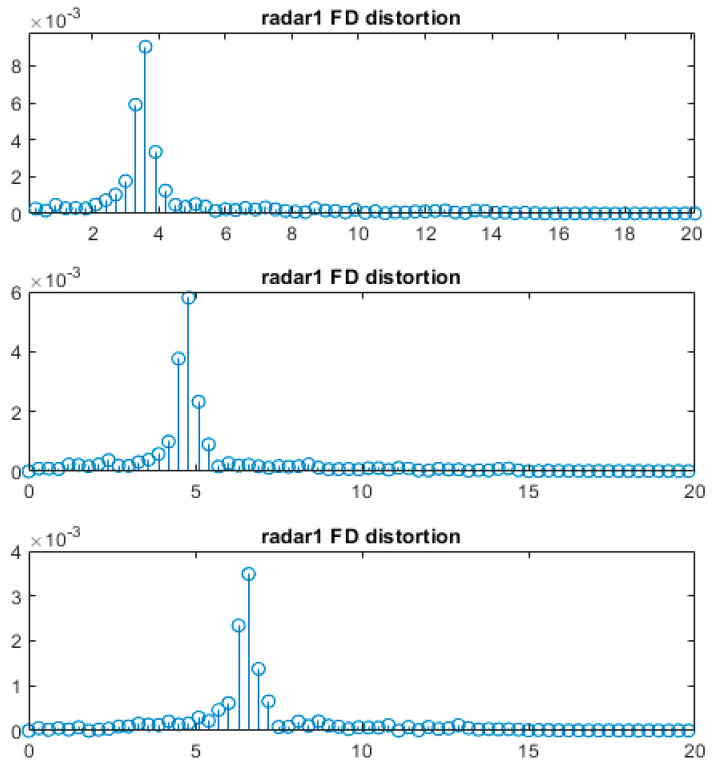
Spectrum for ranges of 3.5 m, 4.7 m, and 6.5 m with a 0.3 m of radar range resolution in a MATLAB simulation. Each axis represents bin numbers and frequency magnitude.

*N* in Equation (6) is the number of FFT points. Figure 3 shows the results of a simulation when *p* is fixed while *n* varies. The experiment environment has a range resolution of γ = 0.3 m, *p* = 0.2 m, and *n* = 11, 15, and 21 in an ideal environment. The most significant ratios between the two peaks were about 82%, 83%, and 85%, respectively, and the ratio of the increase and decrease patterns were revealed through a simple experiment. 

### 3.1. Frequency Shift

**Figure 4:**
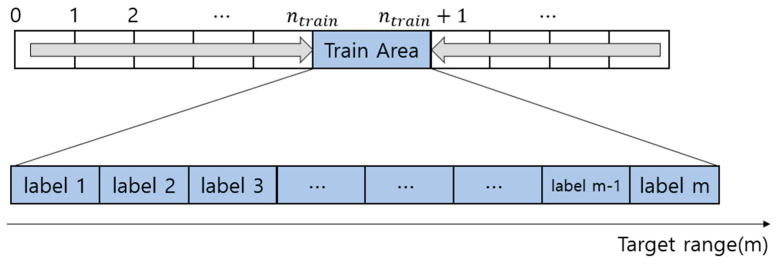
Training area and labels.

Equation (8) demonstrates the independence of the target’s fractional multiple range from *n*. Therefore, the proposed approach focuses solely on learning for the fractional multiple range (p). The term R of $d_{target}$ can be frequency-shifted for an arbitrary integer ($n_{train}$). It serves as the reference value for a frequency shift. Equations (9)–(11) show frequency shift calculation. $n_{max}$ is the peak index or CFAR detected index. $n_{diff}$ is the difference between $n_{train}$ and $n_{max}$. $f_{diff}$ is the frequency difference multiplied by the bin step $\frac{f_{s}}{N}$, and *N* denotes the number of FFT points. Figure 4 illustrates the frequency shift using a diagram. The diagram indicates that it is composed of the number of *m* labels, in which each label represents the distance index with the distance resolution of the bin interval ($\frac{f_{s}}{N}$) divided by *m*. When the beat frequency signal is moved by the frequency shift, all instances of *n* become $n_{train}$.

(9) $$n_{diff}=n_{train}-n_{max}$$

(10) $$f_{diff}=\frac{f_{s}}{N}*n_{diff}$$

(11) $$S_{f}\left(n-n_{diff}\right)=S_{beat}\left(f-f_{diff}\right)$$

### 3.2. Power Normalization and Noise Floor Cancellation

**Figure 5:**
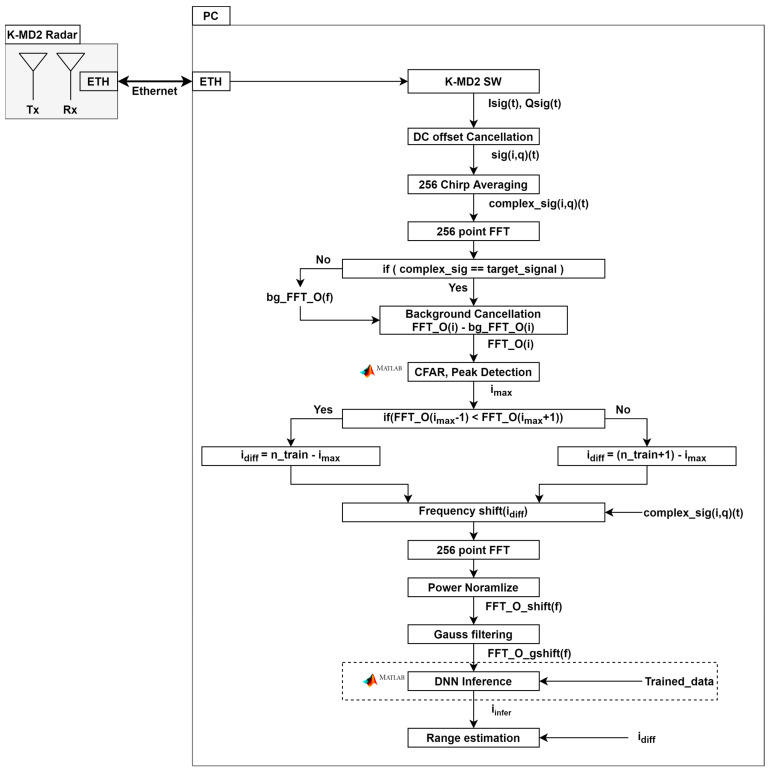
Proposed algorithm flow.

Power normalization is performed to cover the path loss according to the frequency shift. Path loss compensation should be achieved through max peak normalization, avoiding the use of a relational expression. Training data are obtained by measuring within the training region shown in Figure 5. The frequency-shifted signal differs in magnitude from the training data, primarily due to path loss. Power normalization involves dividing the signal by its maximum magnitude. The inference data perform power normalization to mitigate the impact of path loss. This normalization process is applied to the training data to match the main lobe’s magnitude between the two signals. Peak normalization allows the pattern to focus on the sidelobes, which imply a fractional times *p* in Equation (6). In addition, noise floor cancellation reduces the impact of the environment by removing background noise from the target’s spectrum [3]. In the training and inference phase of the neural network model, Gaussian filters of range resolution size were used to mitigate the effects of integer multiples [12].

### 3.3. Deep Learning Architecture

#### 3.3.1. Architecture

**Figure 6:**
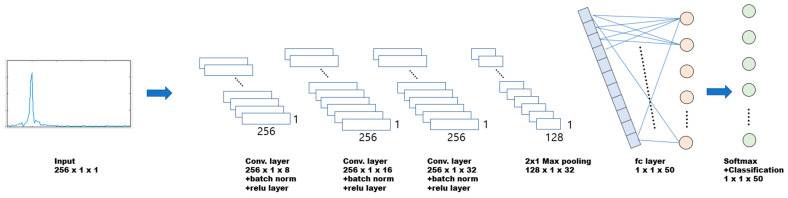
Proposed CNN algorithm.

In this paper, the neural network of the FMCW radar is based on a convolutional neural network (CNN). Figure 6 illustrates the proposed CNN architecture designed for inferring radar labels. The input layer utilizes a power-normalized 256-point spectrum. The convolutional layer incorporates 8, 16, and 32 1 × 1 mask filters for each respective layer [13,14,15,16,17,18]. Batch normalization is performed after each convolutional layer, followed by the rectified linear unit activation function [19,20]. The pooling layer has a 1 × 1 pool size and a stride of 2 [21]. Subsequently, the data are fully connected to the *m* neurons [22,23]. Each neuron calculates the probability for each label via SoftMax, and the reference label is output through the classification layer.

#### 3.3.2. Data Augmentation

**Figure 7:**
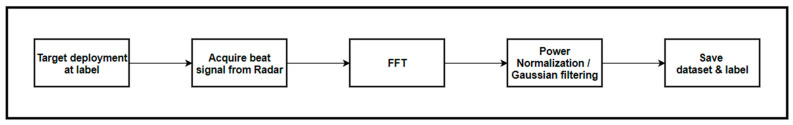
Flow to make a training dataset.

**Figure 8:**
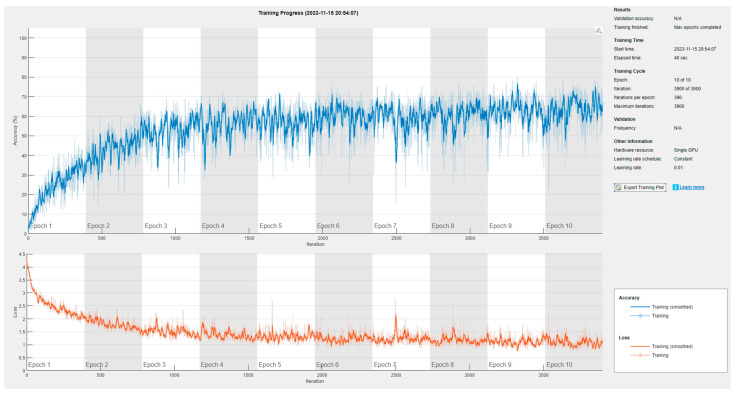
CNN training result.

Training data were created using MATLAB simulations. The range resolution was 0.3 m, $n_{train}$ was 20, *m* was 50, and $label_{res}$ was 0.006 m. The position and label of the target had a 1:1 correspondence. The training data added a random range corresponding to 10% of the label range. A thousand data points were used per label. In assuming an indoor environment, the Rician multipath and additive white Gaussian noise were added. Phase error was introduced to the simulation to emulate real-world conditions, with an offset of approximately ±20 ppm from the carrier frequency [2]. Training was conducted using MATLAB, resulting in a training accuracy of 60% and a loss of 1. The accuracy of the label, however, does not show the distance error, and the mean average error was obtained through experiments. Figure 7 and Figure 8 show how to gather the training dataset and the training results, respectively.

#### 3.3.3. Inference and Restore Integer Range

The label output from CNN is converted to distance through Equation (12). $range_{algo}$ is the range that has been improved by the proposed algorithm.

(12) $$range_{algo}=\left(n_{tarin}+\frac{i_{infer}}{m}-i_{shift}\right)\gamma$$

## 4. Experiments

### 4.1. Environment

**Figure 9:**
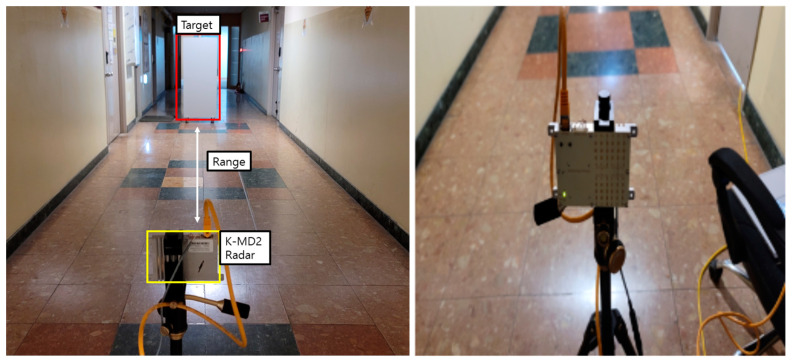
Test environment.

Measurements were conducted in a hallway at Sejong University to validate the proposed algorithm in a real-world setting. The employed radar was an RF-beam K-MD2 FMCW radar, starting at a frequency of 24 GHz with a swept bandwidth of 500 MHz and a range resolution of 0.3 m given in Table 1 [24]. The Gaussian filter used had a 3 dB bandwidth in the impulse response of 1.48 MHz, with an impulse length of 8 samples per symbol and 17 sample points [25,26]. A total of 50,000 training datasets were employed, with each dataset comprising 1000 data points for each label. Figure 9 illustrates the experimental environment. The experiment was conducted by varying the target position within the range of 3 m to 16 m.

### 4.2. Experiment Result

**Figure 10:**
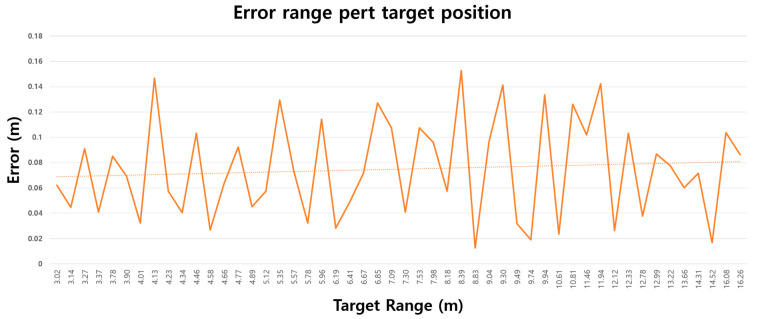
Range estimation results on real targets experiments. (the dotted line = average).

Figure 10 depicts the outcomes obtained by applying the proposed algorithm to the experimental data utilizing real radar measurements. The *x*-axis represents the distance between the target and the radar, while the *y*-axis illustrates the disparity between the measured and actual values. The ground truth was acquired through a laser measuring instrument with a precision of ±2 mm. The mean average error (MAE) given in Equation (13) was determined to be 0.07 m.

(13) $$MAE=\frac{1}{S}\sum_{\;i}^{\;S}E_{i}$$

*E* denotes the range errors between the ground truth and the proposed algorithm’s range, while *S* represents the number of tests conducted. Table 2 displays the performance comparison with IBDE. The range precision achieved by the proposed method was 0.06 m, calculated as 0.3 m divided by 50. CMAE is calculated by multiplying MAE with CR (compensate rate). CR is an index designed to compensate for range resolution, as per Equation (14), where ${Ref}_{bw}$ represents the bandwidth of the reference radar, and ${tar}_{bw}$ is the bandwidth of the target radar [27,28]. The CMAE serves as an index that adjusts for the MAE based on the radar’s range resolution, facilitating a comparison of the algorithm’s improvement rate. The proposed algorithm has advantages in label spacing compared to related works [7,13,29]. The experimental results demonstrate the effectiveness of the proposed algorithm within the 3–16 m range. The experiments were conducted at intervals of approximately 10 cm, offering more refined measurements compared to earlier studies [7,13,29]. Equation (14) was formulated for analyzing the performance and experimental results.

FFT bin spacing refers to the distance between the bins of a signal obtained by performing an n-point FFT on a beat frequency. The bin represents the target location more accurately than the label. If the mean absolute error (MAE) is less than the bin spacing, the bin can be considered to be precisely pointing to the target. Conversely, if the MAE is greater than the bin spacing, the bin is not correctly pointing to the target. Range precision must be determined by taking the MAE into account. In CASE2, the position of the target is accurately expressed rather than the label spacing. Similarly, if the MAE is smaller than the label spacing, the label accurately indicates the target, and in the opposite case, the range precision should be determined by considering the MAE. According to Equation (15), the range precision of the algorithm was 0.07 m.

(14) $$CMAE=MAE*CR\;\;(CR=\frac{Tar_{bw}}{Ref_{bw}})$$

(15) $$range\;precision\left(m\right)=MAX\{MIN\{FFT\;bin\;spacing(m),\;\;label\;spacing(m)\},\;MAE(m)\}$$

In Equation (15), *range precision*(*m*) can be simplified depending on the relations between *FFT bin spacing*(*m*) and *label spacing*(*m*) as the following:

(16) $$\mathrm{range}\;\mathrm{precision}(m)=\left\{\begin{array}{cc} MAX\left\{FFT\;bin\;spacing\left(m\right),MAE\left(m\right)\right\}, & when\;FFT\;bin\;spacing(m)<label\;spacing(m)\\ MAX\left\{label\;spacing\left(m\right),MAE\left(m\right)\right\}, & otherwise \end{array}\right.$$

## 5. Conclusions

This paper addressed the challenge posed by the increasing number of labels resulting from the expansion of the operating range by employing a frequency shift. It introduced a methodology that achieves a broad operating range and high range precision relative to the number of labels. Additionally, the algorithm mitigated clutter by eliminating the noise floor in the frequency domain. The algorithm also reused the training complexity of convolutional neural networks (CNNs) specifically for constant sections. Experimental validation of the algorithm was conducted using FMCW radar measurements. Testing utilized a radar with a range resolution of 0.3 m demonstrated a range precision approximately 4.3 times superior to that of a related work [7]. Through a combination of formulas and experiments, this study demonstrated that identical fractional multiple patterns could be applied to different bin positions using just one fractional pattern. This suggested that neural networks trained on the same data could be efficiently reused for diverse target positions, enabling cost-effective hardware implementation. The experimental results underscored the efficiency and utility of the proposed algorithm for commercial radar sensors, where enhancing performance with minimal hardware costs is a critical concern. However, the limitation of this paper lies in conducting verification solely to discover if the proposed methodology is effective in an actual radar environment. Additional research is needed to optimize such various variables as neural architecture, learning data, number of labels, and power normalization depending on the range resolution.

## Figures and Tables

**Table 1:** Experiment parameters.

Center frequency	24 GHz
Sweep bandwidth	500 MHz
Sweep time	6.9 μs
Range resolution	0.3 m
Sampling rate	38.46 MHz
FFT point	256
Inference range unit	0.006 m

**Table 2:** Comparison of experimental results for algorithms.

Algorithm	Range Resolution (m)	Label #	Range (m, m)	Label Spacing (m)	MAE (m)	CR	CMAE (m)	Range Precision
CSP	1	10	[1, 10)	1	0.2923	0.3	0.08769	0.3
Regression	1	10	[1, 10)	1	0.1647	0.3	0.04941	0.3
IBDE	1	10	[1, 10)	1	0.0690	0.3	0.0207	0.3
Prop. method	0.3	50	[3, 16)	0.006	0.0700	1	0.0700	0.07

## Data Availability

Data are contained within the article.
